# Identification and Expression Patterns of Putative Diversified Carboxylesterases in the Tea Geometrid *Ectropis obliqua* Prout

**DOI:** 10.3389/fphys.2017.01085

**Published:** 2017-12-18

**Authors:** Liang Sun, Qian Wang, Qi Wang, Yuxing Zhang, Meijun Tang, Huawei Guo, Jianyu Fu, Qiang Xiao, Yanan Zhang, Yongjun Zhang

**Affiliations:** ^1^Key Laboratory of Tea Quality and Safety Control, Ministry of Agriculture, Tea Research Institute, Chinese Academy of Agricultural Sciences, Hangzhou, China; ^2^State Key Laboratory for Biology of Plant Diseases and Insect Pests, Institute of Plant Protection, Chinese Academy of Agricultural Sciences, Beijing, China; ^3^College of Horticulture and Plant Protection, Yangzhou University, Yangzhou, China; ^4^College of Plant Protection, Anhui Agricultural University, Hefei, China; ^5^College of Life Sciences, Huaibei Normal University, Huaibei, China

**Keywords:** *Ectropis obliqua*, carboxylesterases (CXEs), odorant-degrading enzymes, phylogenetic analyses, expression patterns, fluorescence *in situ* hybridization

## Abstract

Carboxylesterases (CXEs) belong to a family of metabolic enzymes. Some CXEs act as odorant-degrading enzymes (ODEs), which are reportedly highly expressed in insect olfactory organs and participate in the rapid deactivation of ester pheromone components and plant volatiles. The tea geometrid *Ectropis obliqua* Prout produces sex pheromones consisting of non-ester functional compounds but relies heavily on acetic ester plant volatiles to search for host plants and locate oviposition sites. However, studies characterizing putative candidate ODEs in this important tea plant pest are still relatively scarce. In the present study, we identified 35 candidate *EoblCXE* genes from *E. obliqua* chemosensory organs based on previously obtained transcriptomic data. The deduced amino acid sequences possessed the typical characteristics of the insect CXE family, including oxyanion hole residues, the Ser-Glu-His catalytic triad, and the Ser active included in the conserved pentapeptide characteristic of esterases, Gly-X-Ser-X-Gly. Phylogenetic analyses revealed that the EoblCXEs were diverse, belonging to several different insect esterase clades. Tissue- and sex-related expression patterns were studied via reverse-transcription and quantitative real-time polymerase chain reaction analyses (RT- and qRT-PCR). The results showed that 35 *EoblCXE* genes presented a diversified expression profile; among these, 12 *EoblCXEs* appeared to be antenna-biased, two *EoblCXEs* were non-chemosensory organ-biased, 12 *EoblCXEs* were ubiquitous, and nine *EoblCXEs* showed heterogeneous expression levels among different tissues. Intriguingly, two *EoblCXE* genes, *EoblCXE7* and *EoblCXE13*, were not only strongly localized to antennal sensilla tuned to odorants, such as the sensilla trichodea (Str I and II) and sensilla basiconica (Sba), but were also expressed in the putative gustatory sensilla styloconica (Sst), indicating that these two CXEs might play multiple physiological roles in the *E. obliqua* chemosensory processing system. This study provides the first elucidation of CXEs in the chemosensory system of a geometrid moth species and will enable a more comprehensive understanding of the functions of insect CXEs across lepidopteran species.

## Introduction

The sophisticated olfactory system, particularly the peripheral chemical signal coding, is essential for insects to find mates, locate food, and avoid predators (Dweck et al., 2013; Tauxe et al., 2013; Strauch et al., 2014; Li and Liberles, 2015). Biologically important odorants are generally perceived sensitively and specifically in the multiporous sensilla hairs on insect antennae (Meijerink and van Loon, 1999; Pophof et al., 2005; Park et al., 2013; Sun L. et al., 2014). It is well established that at least three major classes of molecules are involved in this process: odorant-binding proteins (OBPs), odorant receptors (ORs), and odorant-degrading enzymes (ODEs). In brief, airborne odorants enter the hydrosoluble sensillum lymph through the sensilla pores, bind to OBPs, activate ORs and trigger signal transduction cascades and olfactory coding; odorants are then rapidly removed from the vicinity of the ORs by ODEs to restore the sensitivity of the sensory neuron (Rützler and Zwiebel, 2005; Vogt, 2005; Pelosi et al., 2006; Leal, 2013).

The highly sensitive odorant signal transduction pathway of insects represents an excellent model that researchers can use to develop new environmentally friendly pest-management strategies through targeting key molecules and screening biologically active compounds for behavioral control. Previous functional reports regarding OBPs and ORs indeed led to the rapid discovery of high-efficiency pest repellents and attractants. For example, compounds that are behaviorally active in the mirid bug *Adelphocoris lineolatus* were successfully screened *via* studies on the interaction between antenna-enriched AlinOBP10 and its putative ligands (Sun et al., 2013). In the aphid alarm pheromone EBF perception pathway, ApisOBP3 and ApisOBP7 as well as ApisOR5 were proven to be potentially crucial targets for aphid repellent screening (Sun et al., 2012; Zhang R. et al., 2017). However, compared with OBPs and ORs, similar reports on ODEs appear to be rare. Given that the rapid degradation of redundant odorants can rescue the sensitivity of odorant sensory neurons, putative genes encoding insect ODEs that are highly expressed in the chemosensory system should be identified, and their potential roles in odorant degradation deserve thorough exploration.

Carboxylesterases (CXEs) belong to the α/β-fold hydrolase superfamily and are widely distributed in insects and other organisms. CXEs commonly include a conserved catalytic triad (Ser-His-Glu) and specifically catalyze the hydrolysis of ester bonds in various substrates (Oakeshott et al., 1999, 2005). Because most insect species, including hemipteran bugs and lepidopteran moths, utilize aliphatic esters as intraspecific sex pheromones and ovipositional stimulants (Ando et al., 2004; Millar, 2005; Pan et al., 2015), many antennae-biased CXEs have been identified, and their activities associated with sex pheromone and odorant degradation have been assessed (Vogt, 2005; Jacquin-Joly and Maïbèche-Coisne, 2009). The first CXE subfamily of ODEs, known as Apol-SE (Vogt and Riddiford, 1981), or ApolPDE (Ishida and Leal, 2005), was isolated from the giant silk moth, *Antheraea polyphemus*. Subsequently, genes encoding putative antennal esterases were cloned and described across insect species using a polymerase chain reaction (PCR) strategy. These genes included two other CXEs, ApolODE and Apol-IE, in *A. polyphemus* (Ishida and Leal, 2002); Mbra-EST from the cabbage armyworm, *Mamestra brassicae* (Maïbèche-Coisne et al., 2004); D-AP1, a honeybee homolog of CXE in *Apis mellifera* L. (Kamikouchi et al., 2004); Slit-EST and Snon-EST from the Egyptian armyworm, *Spodoptera littoralis*, and the Mediterranean corn borer, *Sesamia nonagrioides* (Merlin et al., 2007); and PjapPDE, cloned from the Japanese beetle, *Popillia japonica* (Ishida and Leal, 2008). Furthermore, through expressed sequence tag (EST) and RNA-Seq analyses, diverse CXE genes have been identified from various insect species, such as *Epiphyas postvittana* (Jordan et al., 2008), *Spodoptera littoralis* (Durand et al., 2010b), *Agrotis ipsilon* (Gu et al., 2013), *Sesamia inferens* (Zhang Y. N. et al., 2014), *Chilo suppressalis* (Liu et al., 2015; Xia et al., 2015), and *Spodoptera litura* (Zhang et al., 2016).

Convincing evidence obtained through biochemical characterization and enzyme kinetic activity analyses showed that Apol-SE/ApolPDE displays expression specific to male antennal sensilla and exhibits rapid catalytic activity toward the acetate sex pheromone component E6Z11-16:OAc (Vogt et al., 1985; Prestwich et al., 1986; Klein, 1987; Ishida and Leal, 2005). *In vitro* functional analyses and potential hydrolyzed substrates of CXEs have also been documented in other insect species, particularly lepidopteran moths, whose main sex pheromone components are acetate esters (Ishida and Leal, 2008; Durand et al., 2010a, 2011; He et al., 2014a,b,c, 2015). Additionally, an extracellular carboxylesterase esterase-6 (EST-6) of *Drosophila melanogaster* has been demonstrated to be a potential ODE for both the sex pheromone ester *cis*-vaccenyl acetate (CVA) and other bioactive volatile esters, such as pentyl acetate (Chertemps et al., 2012, 2015). All of the available data support potential roles of CXEs in degrading either sex pheromones or host plant odorants containing ester functional groups.

The tea geometrid *Ectropis obliqua* Prout is a common pest of the tea plant, *Camellia sinensis* (L.), and causes serious economic damage to tea production (Ye et al., 2014; Zhang G. H. et al., 2014). Multiple electrophysiological and behavioral studies show that *E. obliqua* relies heavily on chemical cues to locate host plants, oviposition sites and conspecific mates. Furthermore, larval infection of tea plants strongly induces the release of several kinds of host volatiles with ester functional groups, and these ester compounds can in turn regulate the ovipositional preference of *E. obliqua* adult females (Sun X. L. et al., 2014). Hence, studies on the molecular mechanism of ester odorant degradation are particularly important for the identification of potential target genes mediating oviposition signal inactivation and the development of ODE-based strategies in geometrid pest management.

In this study, we identified putative genes encoding CXEs by analyzing the BLASTX annotations of transcriptomic data. The phylogenetic relationships between the candidate CXEs and homologs in other Lepidoptera species were further analyzed. Finally, the tissue expression patterns of the identified CXEs were investigated in olfactory organs (particularly in the different antennal sensilla) and non-olfactory organs, and potential functional differentiation was discussed.

## Materials and methods

### Insect rearing and tissue collection

The tea geometrid *E. obliqua* was collected from the Yuhang tea plantation in Zhejiang Province, China. Phylogenetic identity analysis and laboratory colony construction were performed according to Zhang G. H. et al. (2014). The pupae were sexed, and male and female individuals were raised separately until eclosion. Adult moths of different sexes were maintained in different cages and fed a 10% honey solution on water-soaked cotton.

For the tissue-specific expression profile analysis of *E. obliqua* adults, approximately 500 antennae, three abdomens, and 300 legs of both male and female adults 1–3 days after emergence were dissected and collected. Two biological replicates were prepared for RT-PCR, and two additional biological replicates were prepared for qRT-PCR. All of the specimens were immediately stored at −80°C until use.

### RNA extraction and cDNA synthesis

Total RNA from each specimen was extracted with the TRIzol reagent (Invitrogen, Carlsbad, CA, USA) according to the manufacturer's protocol. The integrity of the total RNA was examined through 1.2% agarose electrophoresis, and the purity was assessed using a NanoDrop™ instrument (Wilmington, DE, USA). First-strand cDNA was synthesized from 2 μg of RNA using a FastQuant RT kit with gDNA Eraser (TianGen, Beijing, China) according to the manufacturer's instructions.

### Identification of candidate EoblCXEs and sequence analysis

Candidate EoblCXEs were identified through keyword screening of the BLASTX annotations of transcriptomic data from adult *E. obliqua* chemosensory organs, including the antennae, legs, wings and proboscises. The TBLASTN program was also applied using the previously identified *S. littoralis* CXEs (Durand et al., 2010b) as the query. The open reading frames (ORFs) of genes were predicted using ORF finder (http://www.ncbi.nlm.nih.gov/gorf/gorf.html). The theoretical isoelectric points and molecular weights of the deduced proteins were calculated using the ExPASy tool (http://web.expasy.org/compute_pi/). Homology searches were performed with BLAST (http://blast.ncbi.nlm.nih.gov/). Catalytic residues were predicted by searching the NCBI Conserved Domain Database (http://www.ncbi.nlm.nih.gov/structure/cdd/cdd.shtml). Putative N-terminal signal peptides were predicted using the SignalP 4.0 program (http://www.cbs.dtu.dk/services/SignalP/) (Brunak et al., 2010).

### Phylogenetic analysis

The amino acid sequences of EoblCXEs and CXEs from other species were aligned using ClustalX 2.0 (Larkin et al., 2007). A neighbor-joining tree was constructed using the program MEGA 6.0 with the Jones–Taylor–Thornton (JTT) amino acid substitution model (Tamura et al., 2013). Node support was assessed using a bootstrapping procedure with 1,000 replicates, uniform rates, and pairwise deletion of data gaps. The protein names and accession numbers corresponding to the genes used for construction of the phylogenetic tree are listed in Table S1.

### Reverse-transcription PCR

The tissue-specific expression of *EoblCXEs* was determined via reverse-transcription PCR (RT-PCR) using ExTaq DNA polymerase (TaKaRa, Dalian, China). The *E. obliqua* glyceraldehyde-3-phosphate dehydrogenase (*EoblGAPDH*, GenBank accession no. KT991373) reference gene was employed as an internal control to normalize target gene expression in order to correct for sample-to-sample variation. The specific primers used for amplification are listed in Table S2.

The experiment was performed according to a previous report (Sun et al., 2017b): each reaction of 50 μL contained 1 μL of 200 ng/μL (200 ng) single-stranded cDNA, 5 μL of 10 × ExTaq buffer, 4 μL of deoxyribonucleoside triphosphates (dNTPs), 2 μL of each primer and 0.25 U of ExTaq DNA polymerase. The PCR conditions were as follows: initial denaturation at 94°C for 4 min followed by 40 cycles of 94°C for 30 s, 55–65°C for 30 s, and 72°C for 30 s and a final elongation step at 72°C for 10 min. After PCR, the products were analyzed in 1.5% agarose gels. To check reproducibility, each RT-PCR run for each sample was performed with two biological replicates and three technical replicates. The relative expression levels of the *EoblCXE* genes in different tissues were calculated using the ratio of RT-PCR band intensity between the target gene and the internal reference gene, *EoblGAPDH*, using Bio-Rad Quantity One 4.6.2 software (Zhang et al., 2013).

### Quantitative real-time PCR

Based on the RT-PCR results, 18 *EoblCXEs* were randomly selected to conduct quantitative real-time PCR (qRT-PCR). The experiment was performed using an ABI 7500 Real-Time PCR System (Applied Biosystems, Carlsbad, CA, USA), and each reaction was conducted in a 20-μL reaction mixture containing 10 μL of 2 × SYBR Green PCR Master Mix (TaKaRa, Dalian, Liaoning, China), 0.8 μL of each primer (10 μM), 0.4 μL of ROX Reference Dye II, 2 μL of sample cDNA (200 ng), and 6.0 μL of sterilized H_2_O. The qPCR cycling parameters were as follows: 95°C for 30 s followed by 40 cycles of 95°C for 5 s and 60°C for 31 s. Subsequently, the fluorescence was measured using a 55–95°C melting curve to detect a single gene-specific peak and to confirm the absence of primer dimer peaks; single, discrete peaks were detected for all primers tested.

The primers employed for qPCR (Table S3) were designed using the Beacon Designer 7.90 program (PREMIER Biosoft International). The reference gene *EoblGAPDH* was found to be expressed at a similar level in different tissues and was used as an internal control to normalize target gene expression in order to correct for sample-to-sample variation (Sun et al., 2017a). The amplification efficiency for the target and reference genes was assessed using gradient dilution templates to examine the variation of ΔC_T_ (C_T_, _Target gene_ − C_T, reference gene_) with template dilution (Livak and Schmittgen, 2001). The absolute values of the slopes of all lines obtained from template dilution plots (log cDNA dilution vs. ΔC_T_) were close to zero, indicating that the efficiency for *EoblCXEs* was similar to that for *EoblGAPDH*. Non-template reactions (replacing cDNA with sterilized H_2_O) were performed as negative controls. To check the reproducibility of the qPCR assays, each reaction for each sample was performed with three technical replicates and two biological replicates.

Comparative analyses of target gene expression between different tissues were performed using one-way nested analysis of variance (ANOVA) followed by Tukey's honestly significant difference (HSD) test. The relative mRNA expression levels between males and females of 10 antennae-biased *EoblCXEs* were compared with Student's *t*-test. All analyses were performed using SPSS Statistics 18.0 software (SPSS Inc., Chicago, IL, USA).

### Fluorescence *in situ* hybridization

Based on the observed tissue expression patterns and the results of phylogenetic analyses, two *EoblCXE* genes, *EoblCXE*7, and *EoblCXE13*, were selected for fluorescence *in situ* hybridization assays. Biotin-labeled antisense or sense RNA probes were transcribed from the linearized recombinant pGEM-T vector using a biotin RNA Labeling Mix (SP6/T7) (Roche, Mannheim, Germany) following the recommended protocols. RNA probes were subsequently fragmented to an average length of approximately 400 bp via incubation in carbonate buffer (80 mM NaHCO_3_, 120 mM Na_2_CO_3_, pH 10.2).

The experiment was performed following a reported protocol (Wang et al., 2017). The antennae of both male and female 1–3-day-old moths were dissected, embedded with Tissue-Tek optimal cutting temperature (O.C.T.) compound (Sakura Finetek, Torrance, CA, USA) and rapidly frozen at −60°C. Sections (12 μm) were prepared using a Cryostar NX50 cryostat (Thermo Scientific, San Jose, CA, USA) at −20°C, thaw-mounted on SuperFrost Plus microscope slides (Fisher Scientific, Pittsburgh, PA, USA), and air-dried at room temperature for 15 min. After a series of fixing and washing procedures, the tissue sections were covered with 100 μL of hybridization solution containing biotin-labeled antisense RNA probes and incubated at 60°C for at least 16 h. After hybridization, the slides were washed twice for 20 min in 0.2 × saline-sodium citrate (SSC) at 60°C and treated with 1% blocking reagent (Roche, Basel, Switzerland) in TBST for 30 min at room temperature. Biotin-labeled probes were detected via incubation with streptavidin-horseradish peroxidase (HRP) (Perkin Elmer, Boston, MA, USA) diluted 1:100 in TBS with 0.03% Triton X-100 and 1% blocking reagent at 37°C for 1 h. After three 5-min washes in TBS with 0.05% Tween 20 (Sigma, Louis, MO, USA), the biotin-labeled probes were detected with the TSA Plus Fluorescein System (Perkin Elmer). Images were captured via laser scanning microscopy (LSM) using a Zeiss LSM880 confocal microscope (Zeiss, Oberkochen, Germany). Photoshop CS5 (Adobe Systems, San Jose, CA, USA) was employed to adjust the brightness or contrast of the figures.

## Results

### Identification and sequence characteristics of candidate EoblCXEs

Thirty-five candidate EoblCXEs were identified in the chemosensory organs of *E. obliqua*. Of these 35 candidates, 28 EoblCXEs possessed full-length open reading frames (ORFs), and seven lacked either the 5′ or 3′ region (Table 1). The candidate sequences were designated EoblCXE1-35 according to their presumptive orthologs in other insects, particularly *S. littoralis, S. exigua* and *S. inferens*, and were deposited in the GenBank database under sequential accession numbers from KX015843 to KX015877 (Table 1).

**Table 1:** BLASTX hits for candidate CXEs identified in the chemosensory organs of *E. obliqua* adults.

**Gene name**	**Acc. number**	**ORF**	**Complete**	**Best BLASTX hit**				
		**(Aa)**	**ORF**					
				**Name**	**Species**	**Protein ID**	***E*-value**	**Identity (%)**
*EoblCXE1*	KX015843	565	Yes	Odorant degrading enzyme CXE1	*Sesamia inferens*	AII21978.1	0	69
*EoblCXE2*	KX015844	519	Yes	Odorant degrading enzyme CXE6	*Sesamia inferens*	AII21982.1	7.00E-140	46
*EoblCXE3*	KX015845	536	Yes	Odorant degrading enzyme CXE3	*Operophtera brumata*	KOB64827.1	0	64
*EoblCXE4*	KX015846	519	Yes	Esterase	*Sesamia nonagrioides*	ABH01082.1	8.00E-147	46
*EoblCXE5*	KX015847	596	Yes	Venom carboxylesterase-6-like	*Amyelois transitella*	XP_013187979.1	0	63
*EoblCXE6*	KX015848	559	Yes	Carboxylesterase ae27	*Operophtera brumata*	KOB73502.1	0	69
*EoblCXE7*	KX015849	510	3′ lack	Carboxylesterase, partial	*Operophtera brumata*	KOB58168.1	0	57
*EoblCXE8*	KX015850	557	3′ lack	Antennal carboxylesterase 14	*Chilo suppressalis*	AKS40366.1	0	53
*EoblCXE9*	KX015851	559	Yes	Antennal esterase CXE9	*Spodoptera littoralis*	ACV60236.1	0	62
*EoblCXE10*	KX015852	533	Yes	Antennal esterase CXE10	*Spodoptera exigua*	AEJ38207.1	0	59
*EoblCXE11*	KX015853	524	Yes	Esterase FE4-like	*Papilio machaon*	XP_014362170.1	8.00E-176	49
*EoblCXE12*	KX015854	556	Yes	Para-nitrobenzyl esterase-like	*Amyelois transitella*	XP_013193079.1	0	57
*EoblCXE13*	KX015855	561	Yes	Carboxylesterase	*Ostrinia furnacalis*	BAR64778.1	0	71
*EoblCXE14*	KX015856	366	5′/3′ lack	Antennal esterase CXE14	*Operophtera brumata*	KOB70764.1	3.00E-131	53
*EoblCXE15*	KX015857	493	5′ lack	Antennal esterase CXE15	*Spodoptera littoralis*	ACV60242.1	1.00E-149	53
*EoblCXE16*	KX015858	564	Yes	Antennal carboxylesterase 10,	*Chilo suppressalis*	AKS40362.1	0	59
*EoblCXE17*	KX015859	546	Yes	Antennal esterase CXE17	*Spodoptera litura*	ADR64699.1	0	62
*EoblCXE18*	KX015860	543	Yes	Carboxylesterase CarE-10	*Operophtera brumata*	KOB69769.1	0.00E+00	68
*EoblCXE19*	KX015861	610	Yes	Antennal esterase CXE19	*Spodoptera littoralis*	ACV60246.1	0	79
*EoblCXE20*	KX015862	545	Yes	Antennal carboxylesterase 17	*Chilo suppressalis*	AKS40369.1	0.00E+00	51
*EoblCXE21*	KX015863	541	Yes	Antennal esterase CXE14	*Spodoptera exigua*	AEJ38205.1	0	63
*EoblCXE22*	KX015864	569	Yes	Carboxylesterase	*Operophtera brumata*	KOB70767.1	0	65
*EoblCXE23*	KX015865	586	Yes	Juvenile hormone esterase-like	*Plutella xylostella*	XP_011557003.1	1.00E-95	37
*EoblCXE24*	KX015866	568	Yes	Esterase FE4-like isoform X2	*Bombyx mori*	XP_012546670.1	0	52
*EoblCXE25*	KX015867	568	3′ lack	Esterase FE4-like isoform X2	*Bombyx mori*	XP_012546670.1	0.00E+00	56
*EoblCXE26*	KX015868	524	Yes	Antennal esterase CXE12	*Cydia pomonella*	AMB19665.1	0.00E+00	67
*EoblCXE27*	KX015869	535	Yes	Odorant degrading enzyme CXE3	*Operophtera brumata*	KOB64827.1	0	64
*EoblCXE28*	KX015870	516	5′ lack	Odorant degrading enzyme CXE3	*Operophtera brumata*	KOB64827.1	0.00E+00	62
*EoblCXE29*	KX015871	568	Yes	Odorant degrading enzyme CXE3	*Operophtera brumata*	KOB64827.1	0	62
*EoblCXE30*	KX015872	539	Yes	Odorant degrading enzyme CXE3	*Operophtera brumata*	KOB64827.1	0.00E+00	65
*EoblCXE31*	KX015873	539	Yes	Odorant degrading enzyme CXE3	*Operophtera brumata*	KOB64827.1	0.00E+00	65
*EoblCXE32*	KX015874	706	Yes	Carboxylesterase 3 isoform X1	*Papilio polytes*	XP_013137785.1	0	65
*EoblCXE33*	KX015875	556	Yes	Carboxyl esterase CCE025a	*Helicoverpa armigera*	ADF43491.1	0	74
*EoblCXE34*	KX015876	564	Yes	Antennal esterase CXE9	*Spodoptera littoralis*	ACV60236.1	0	68
*EoblCXE35*	KX015877	194	5′ lack	Odorant degrading enzyme CXE9	*Sesamia inferens*	AII21983.1	1.00E-92	68

The amino acid identity among the 35 candidate EoblCXEs ranged from 13 to 98% (Table S4). The 28 full-length EoblCXEs exhibited an average coding region length of 1650 bp and encoded 519 to 706 amino acids. Their predicted theoretical isoelectric points ranged from 4.95 to 8.80, and their calculated molecular masses ranged from 58.10 to 74.89. Putative N-terminal signal peptide prediction showed that 16 of the 28 sequences displayed typical sequence cleavage sites. Multiple sequence alignments revealed that all 28 full-length EoblCXEs displayed a conserved sequence motif including the oxyanion hole residues, the catalytic triad (Ser-Glu-His), and the Ser active site in the conserved pentapeptide Gly-X-Ser-X-Gly, characteristic of esterases (Table 2).

**Table 2:** Motif analysis and biochemical characteristics of the 28 putative EoblCXEs with full-length sequences.

	**Catalytic motifs (amino acids)**				**Predicted SP/pI/MW**		
	**Oxyanion hole**	**GxSxG**	**E**	**H**	**SP**	**MW (kDa)**	**pI**
EoblCXE1	GGC	GESAG	+	+	+	63.5	8.32
EoblCXE2	GGG	GESWG	+	+	+	59.5	6.3
EoblCXE3	GGG	GESAG	+	+	−	59.9	5.84
EoblCXE4	GGG	GESWG	+	+	+	58.1	7.99
EoblCXE5	AGG	GCSAG	+	+	+	66.51	6.66
EoblCXE6	AEE	GHSSA	+	+	+	60.94	5.46
EoblCXE9	IGC	GSSSG	+	+	−	64.02	7.88
EoblCXE10	GGG	GESAG	+	+	−	59.72	5.86
EoblCXE11	GGG	GVSAG	+	+	−	58.26	5.64
EoblCXE12	GGA	GYSAG	+	+	+	61.29	4.95
EoblCXE13	GGA	GCSAG	+	+	+	62.17	6.07
EoblCXE16	AGG	GYSAG	+	+	+	60.64	6.76
EoblCXE17	GGG	GESAG	+	+	+	61.25	7.66
EoblCXE18	GGA	GQSAG	+	+	+	61.09	7.97
EoblCXE19	GGG	GHDAG	+	+	+	69.14	5.3
EoblCXE20	GGG	GESAG	+	+	+	60.97	8.52
EoblCXE21	GGA	GGSAG	+	+	+	59.86	5.24
EoblCXE22	GGA	GGSAG	+	+	+	63.57	5.74
EoblCXE23	GGG	GHSTG	+	+	+	66.54	6.55
EoblCXE24	GGA	GESAG	+	+	−	63.57	8.8
EoblCXE26	GGG	GCSAG	+	+	−	59.25	6.02
EoblCXE27	GGG	GESAG	+	+	−	60.08	6.78
EoblCXE29	GGG	GESSG	+	+	+	63.93	5.41
EoblCXE30	GGG	GESAG	+	+	−	60.53	5.86
EoblCXE31	GGG	GESAG	+	+	−	60.6	6.05
EoblCXE32	GGN	GQGSG	+	+	+	74.89	7.91
EoblCXE33	GGG	GESAG	+	+	+	62.33	5.21
EoblCXE34	VGC	GSSSG	+	+	−	63.91	5.55

### Phylogenetic analyses

**Figure 1:**
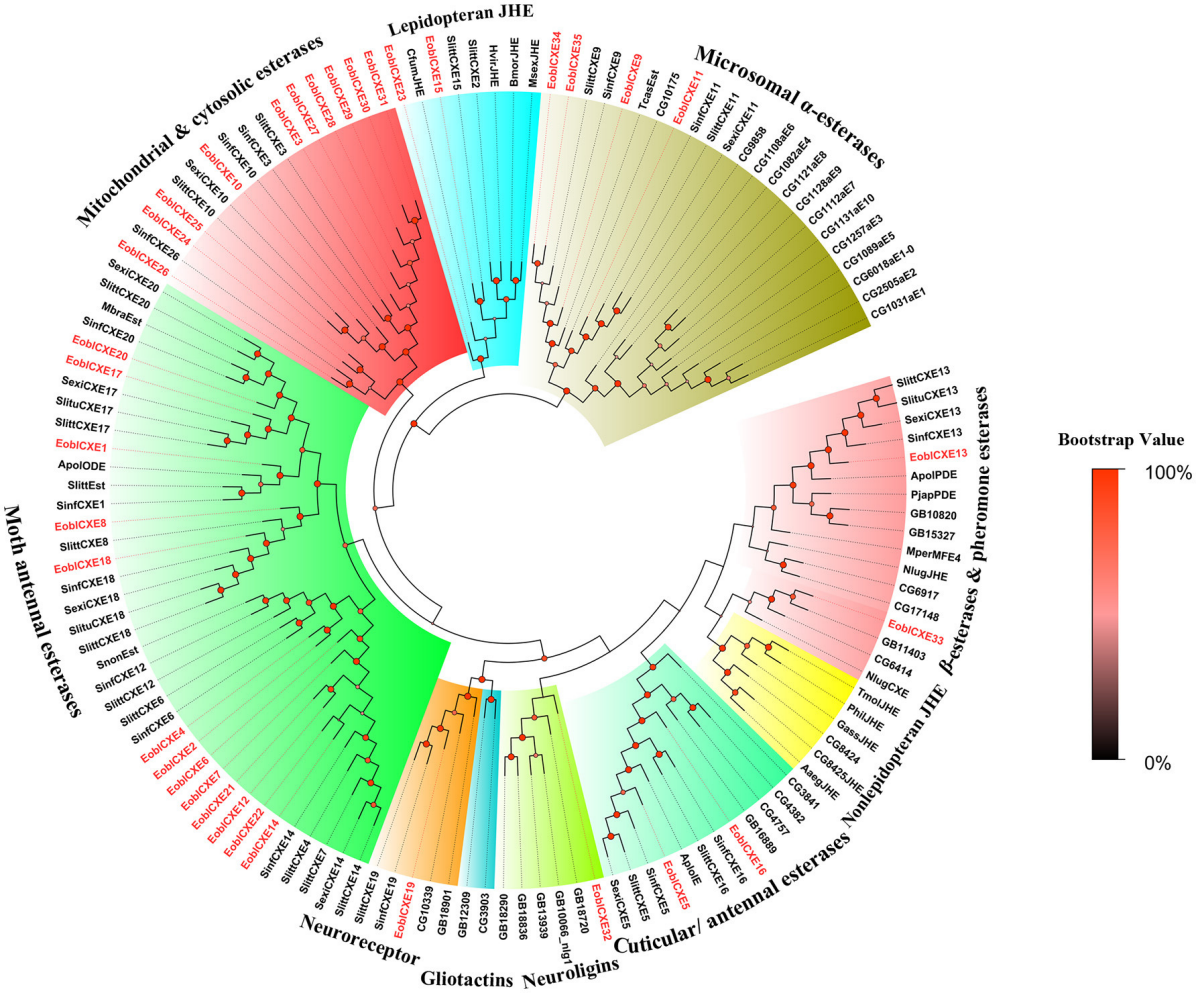
Phylogenetic tree of insect carboxylesterases (CXEs). The tree was constructed with MEGA 6.0 using the neighbor-joining method. The values at the nodes are the results of bootstrapping with 1,000 replicates. EoblCXEs are shown in red. The accession numbers are given in Table S1 and alignment document was included in Supplementary Material.

To define the putative functions of the candidate EoblCXEs, phylogenetic analyses were performed (Figure 1). The results revealed that the insect esterases could be divided into 10 major clades: mitochondrial and cytosolic esterases, dipteran microsomal α-esterases, cuticular and antennal esterases, ß-esterases and pheromone esterases, lepidopteran juvenile hormone esterases (JHE), non-lepidopteran JHE, moth antennal esterases, neuroligins, neuroreceptors, and gliotactins (Oakeshott et al., 2005; Durand et al., 2010b).

EoblCXEs were generally distributed in eight different clades: EoblCXE13 and 33, along with the pheromone-degrading enzymes Apol-PDE and Pjap-PDE, clustered with the ß-esterase and pheromone esterase group; EoblCXE5 and 16 were distributed within a clade of cuticular and antennal esterases; and EoblCXE19, EoblCXE32, two EoblCXEs (EoblCXE15 and 23), four EoblCXEs (EoblCXE9, 11, 34, and 35), and 10 EoblCXEs (EoblCXE3, 10, 24–31) were assigned to neuroreceptors, neuroligins, lepidopteran JHE, dipteran microsomal α-esterases and mitochondrial and cytosolic esterases, respectively. The moth antennal esterases exhibited the greatest abundance of EoblCXEs (13 EoblCXEs, including EoblCXE1, 2, 4, 6–8, 12, 14, 17, 18, and 20–22), whereas no EoblCXEs clustered into the non-lepidopteran JHE or gliotactin clades (Figure 1).

### Tissue- and sex-related expression patterns of candidate *EoblCXE* genes

**Figure 2:**
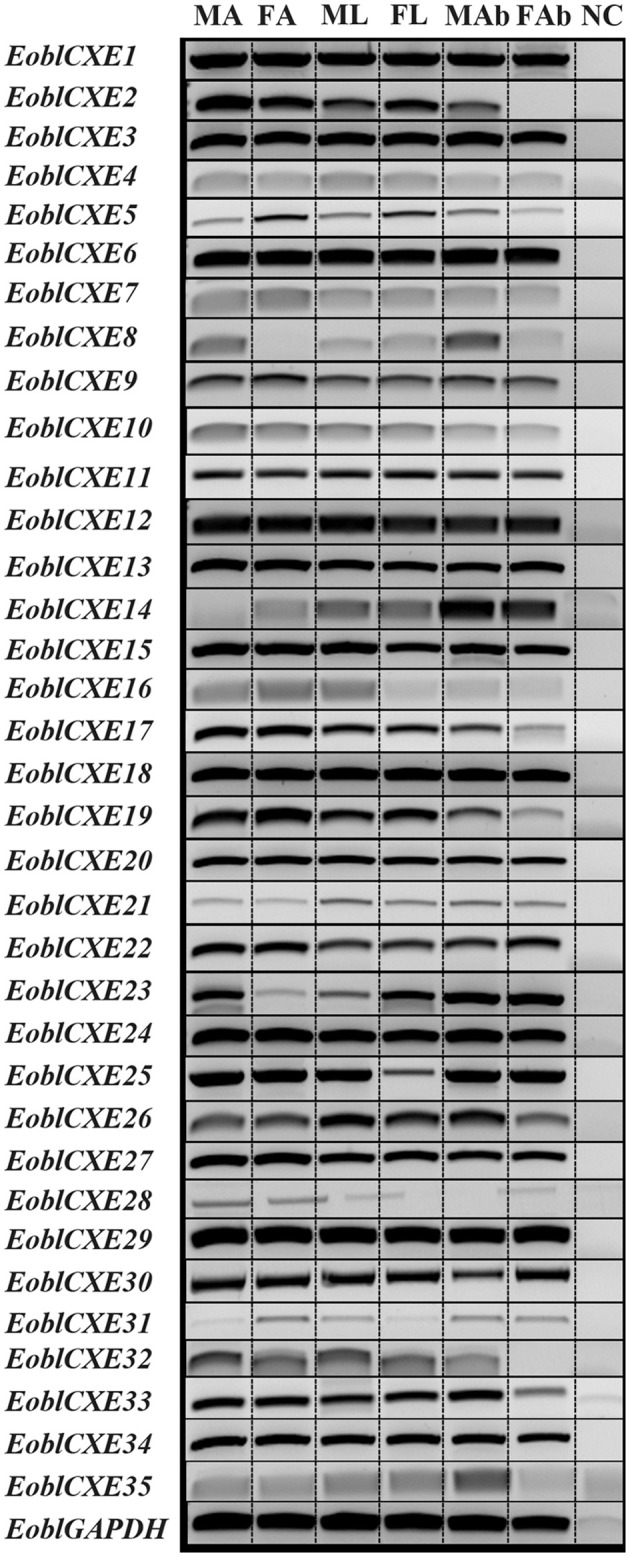
Tissue-related expression patterns of *EoblCXE* genes, as revealed via RT-PCR. The *EoblGAPDH* gene was used as a positive control, and NC (no cDNA template) was used as a negative control. MA, male antennae; FA, female antennae; ML, male legs; FL, female legs; MAB, male abdomen; FAB, female abdomen. The original image of this is shown in Figure S2.

To clarify whether candidate EoblCXEs could function in chemosensory organs with physiological roles in odorant degradation, the tissue- and sex-related expression profiles of the 35 *EoblCXE* genes were determined via RT-PCR. As shown in Figure 2, the *EoblCXE* genes displayed four general expression patterns: 12 *EoblCXE* genes (*EoblCXE2, 5*, 7, *10, 13, 15–17, 19, 20, 22*, and *24*) were strongly expressed in olfactory organ antennae; two *EoblCXEs* (*EoblCXE14* and *23*) were non-chemosensory organ biased; 12 *EoblCXEs* (*EoblCXE1, 3, 4, 6, 9, 11, 12, 18, 27, 29, 30*, and *34*) were ubiquitous, and their expression levels were comparable among the tested tissues; and nine *EoblCXEs* (*EoblCXE8, 21, 26, 35, 25, 28*, and *31–33*) exhibited heterogeneous expression profiles.

**Figure 3:**
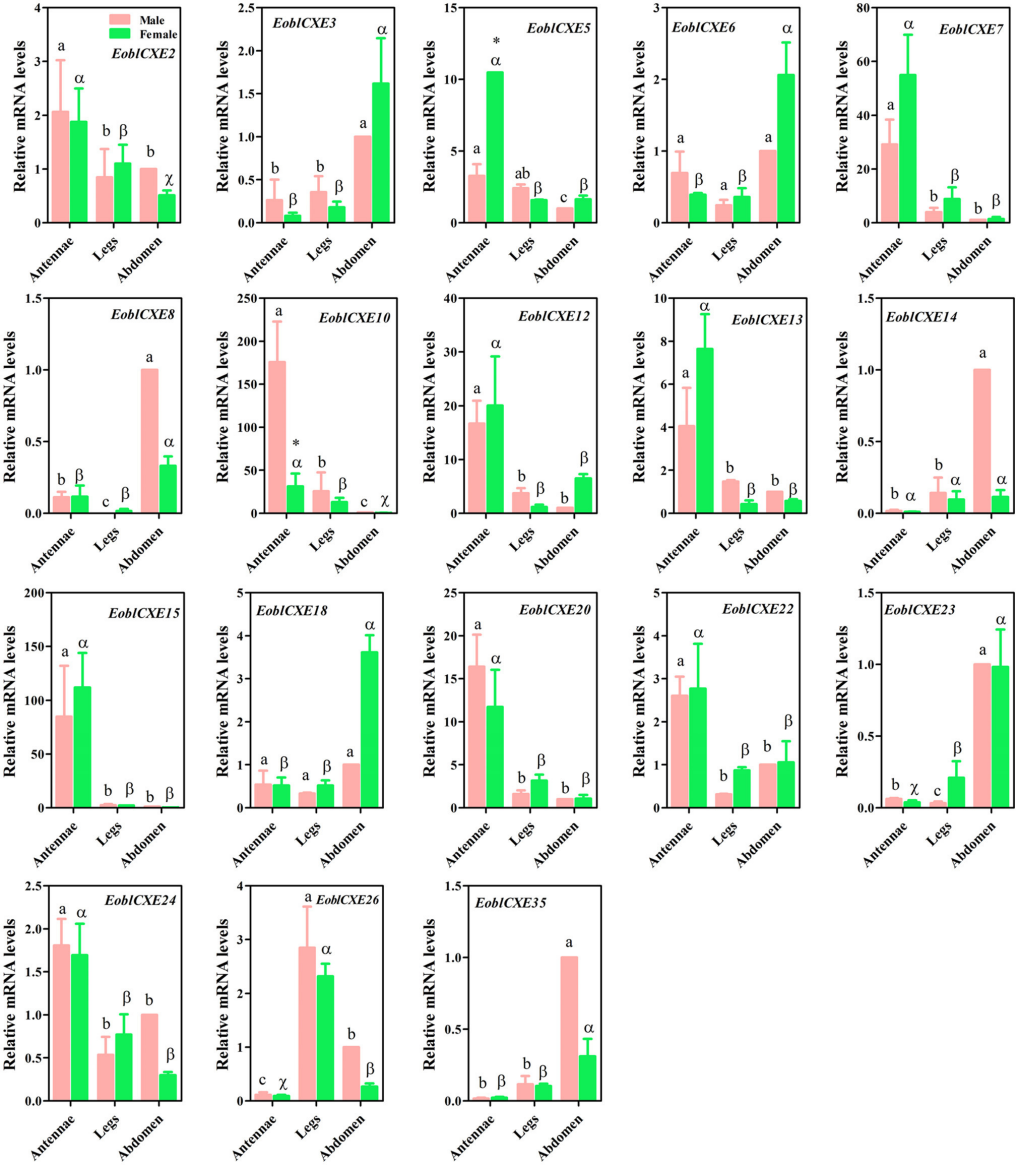
Relative mRNA levels of *EoblCXE* genes in different tissues of male and female *E. obliqua* adults, as revealed via qRT-PCR. The reference gene *EoblGAPDH* was found to be expressed at a similar level in different tissues and was employed as an internal control to normalize target gene expression in order to correct for sample-to-sample variation (Sun et al., 2017a). The amplification efficiency for the target and reference genes were assessed using gradient dilution templates to examine the variation of ΔC_T_ (C_T_, _Target gene_ − C_T, reference gene_) with template dilution (Livak and Schmittgen, 2001). The absolute values of the slopes of all lines from the template dilution plots (log cDNA dilution vs. ΔC_T_) were close to zero, indicating that the efficiency for *EoblCXEs* was similar to that for *EoblGAPDH*. The fold changes are relative to the transcript levels in the male abdomen. The error bars represent the standard error, and different letters (a, b and c for male; α, ß, and χ for females) above each bar denote significant differences (*P* < 0.05). The *t* and *p*-values in Student's *t*-test are shown in Table S5 and the asterisk indicates significantly different relative expression levels between male and female antennae.

To confirm the RT-PCR results, 18 candidate EoblCXE genes randomly selected from all expression patterns were quantified through qRT-PCR assays. The qRT-PCR results are shown in Figure 3. Similar to the RT-PCR results, *EoblCXE2, 5, 7, 10, 13, 15, 20, 22*, and *24* were strongly expressed in moth antennae, whereas *EoblCXE14* and *23* were primarily expressed in the abdomen (a non-chemosensory organ), and *EoblCXE6* was ubiquitously expressed within different tissues. However, the qRT-PCR and RT-PCR results were somewhat contradictory; the qRT-PCR results showed that *EoblCXE3* was highly expressed in the abdomens of both sexes, whereas *EoblCXE12* and *26* were highly expressed in the antennae and legs, respectively.

To dissect the putatively different roles of *EoblCXEs* in olfaction between male and female moths, sex-biased expression profiles were determined for the antennae-enriched *EoblCXE* genes. *Eight* of the 10 tested *EoblCXE* genes (*EoblCXE2, 5, 7, 10, 12, 13, 15, 20, 22*, and *24*) exhibited comparable expression levels between the sexes. In contrast, *EoblCXE5* and *10* were highly expressed in female and male antennae, respectively.

### Cellular localization of *EoblCXE7* and *EoblCXE13* within different antennal sensilla

**Figure 4:**
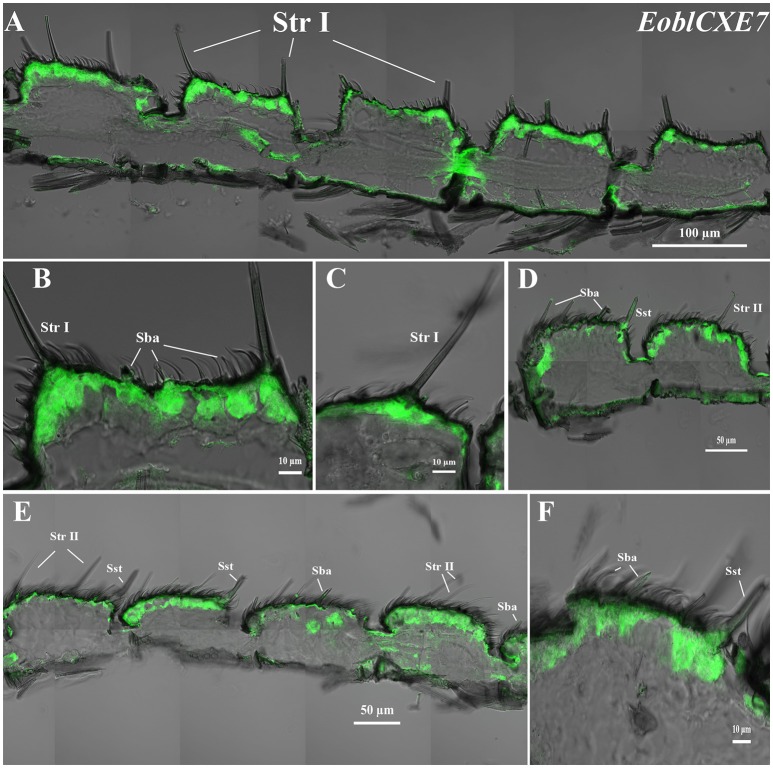
Localization of the *EoblCXE7* gene in different antennal sensilla of both sexes, as revealed via fluorescence *in situ* hybridization. **(A–C)** male antennae; **(D–F)** female antennae; Str I, sensilla trichodea I; Str II, sensilla trichodea II; Sba, sensilla basiconica; Sst, sensilla styloconica.

**Figure 5:**
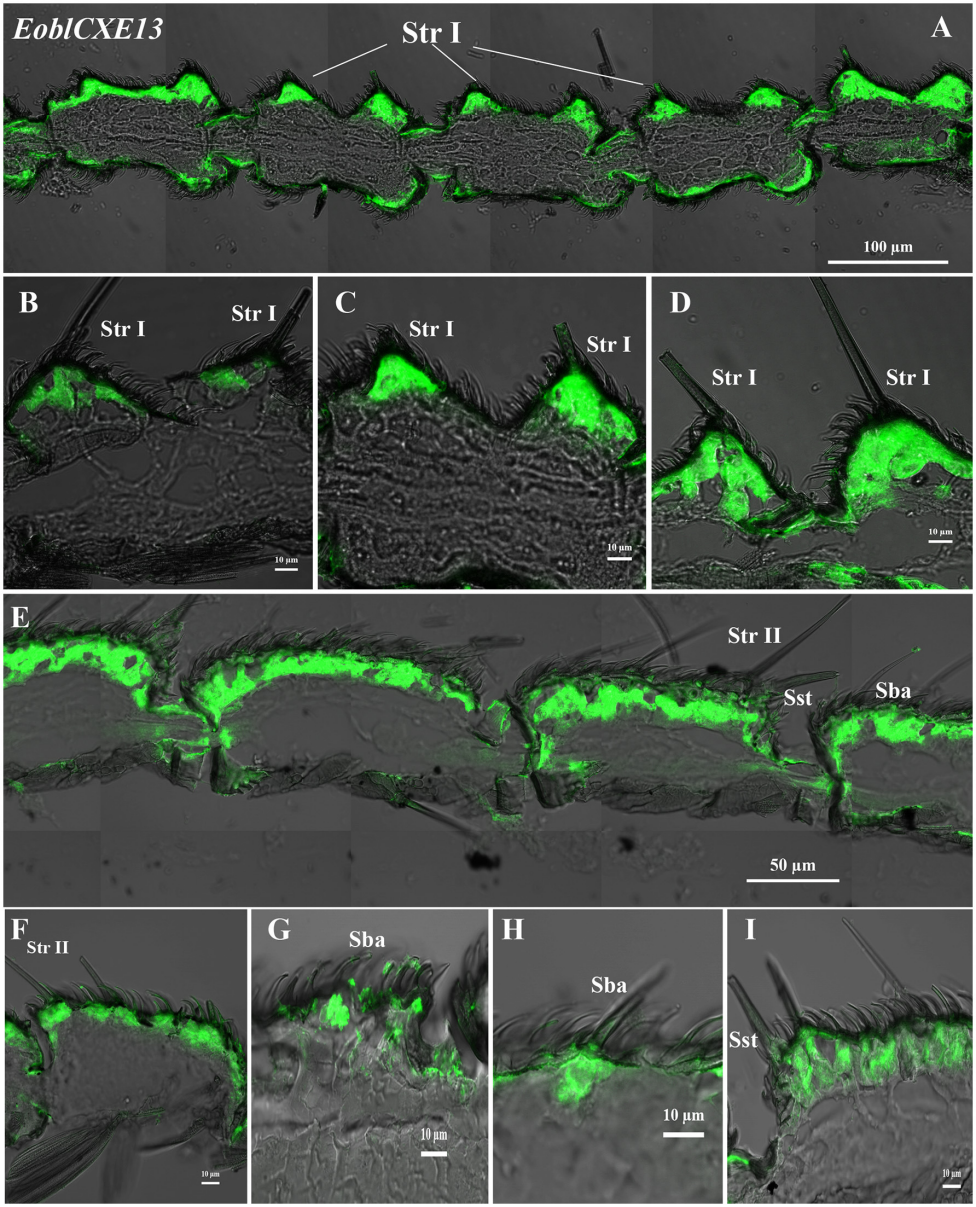
Localization of the *EoblCXE13* gene in different antennal sensilla of both sexes, as revealed via fluorescence *in situ* hybridization. **(A–D)** male antennae; **(E–I)** female antennae; Str I, sensilla trichodea I; Str II, sensilla trichodea II; Sba, sensilla basiconica; Sst, sensilla styloconica.

*EoblCXE7* and *EoblCXE13* were strongly labeled on the sensilla sides of both male and female antennae but differed from each other in their cellular localization in the different types of sensilla found in each sex (Figures 4, 5). *EoblCXE7* exhibited a similar localization in the antennae of male and female moths (Figures 4A–F). The antisense *EoblCXE7* probe clearly labeled the base of the sensilla trichodea (Str I for male and Str II for female moths), sensilla basiconica (Sba) and sensilla styloconica (Sst) (Figures 4A–C). The scale sides of the antennae were not labeled in either sex. Compared with *EoblCXE7, EoblCXE13* showed different expression patterns between male and female antennal sensilla (Figures 5A–I). The antisense *EoblCXE13* probe was restricted to the base of Str I rather than Sba or Sst in male moths (Figures 5A–D). In contrast, strong labeling was detected not only in Str II but also in Sba and Sst in female moths (Figures 5E–I). The sense probes of *EoblCXE7* and *EoblCXE13* produced no positive signals (Figure S1).

## Discussion

In the present study, we identified and characterized 35 candidate genes encoding EoblCXEs from the chemosensory organs of the moth *E. obliqua* through transcriptomic analysis, including 28 full-length sequences. Phylogenetic analyses and tissue- and sex-related expression profiling showed that EoblCXEs exhibited diverse sequence structures, multiple subfamily clades and distinct expression patterns, suggesting potential differentiation of physiological functions among EoblCXEs. As expected, we demonstrated that 12 *EoblCXE* genes were highly expressed in *E. obliqua* antennae, particularly *EoblCXE7* and *EoblCXE13*, which were strongly localized to the olfactory sensilla of both sexes. These results were consistent with the previously reported proposition that CXEs function specifically in insect olfaction and are involved in olfactory signal termination and maintenance of the sensitivity of the olfactory sensilla (Vogt and Riddiford, 1981; Vogt et al., 1985; Ishida and Leal, 2005; Vogt, 2005; Durand et al., 2010a,b, 2011; Chertemps et al., 2012, 2015).

Gene sequence identification represents the first step in elucidating the potential physiological functions of insect CXEs. Due to the lack of genomic information for *E. obliqua*, we identified putative CXEs through a transcriptomic approach. The number of putative CXE genes (35) identified in *E. obliqua* (Table 1) was comparable to those found in insect species with available genome data, including *D. melanogast*er (35 genes), *A. gambiae* (51 genes), *A. aegypti* (49 genes) and *A. mellifera* (24 genes). However, this number was significantly greater than the number of CXE genes identified in moth species that utilize ester compounds as intraspecific sex pheromones, including 20 from *S. littoralis* (Merlin et al., 2007; Durand et al., 2010b), 24 from *S. litura* (Zhang et al., 2016), 20 from *S. inferens* (Zhang Y. N. et al., 2014), 19 from *C. suppressalis* (Liu et al., 2015), 17 from *A. ipsilon* (Gu et al., 2013), and 30 from *Cnaphalocrocis medinalis* (Zhang Y. X. et al., 2017).

Insect CXEs expressed in the olfactory system are mainly related to ester odorant degradation, particularly that of lepidopteran moth sex pheromones (Vogt and Riddiford, 1981; Vogt et al., 1985; He et al., 2015); however, *E. obliqua* females produce sex pheromones containing unsaturated hydrocarbons and enantiomers of epoxy hydrocarbons rather than acetate esters (Yang et al., 2016a). Thus, it is particularly interesting that the number of CXE genes from *E. obliqua* was significantly higher than that in species that utilize ester compounds as intraspecific sex pheromones. We speculate that this situation might be attributed to either the use of a different sequencing strategy or the fact that *E. obliqua* adults depend on the detection of multiple odorants with ester functional groups to find host plants and egg-laying sites. Our use of a high-throughput RNA-sequencing approach in the chemosensory organs (adult antennae of both sexes, legs, wings and proboscises) enabled us to identify as many CXE genes from *E. obliqua* chemosensory organs as possible. The tea plant, which is the most preferred host of *E. obliqua*, releases large quantities of ester compounds, particularly under attack by *E. obliqua* caterpillars; these ester compounds, such as (*Z*)-3-hexenyl hexanoate and (*Z*)-3-hexenyl acetate, can in turn regulate the host searching and ovipositional preferences of *E. obliqua* female adults (Wang, 2010; Sun X. L. et al., 2014). Hence, we propose that some EoblCXEs might act as candidate ODEs and potentially exhibit crucial physiological functions in the degradation of tea plant volatiles with ester functional groups rather than the degradation of sex pheromone components produced by *E. obliqua* females. This inference corresponds well to the tissue- and sex-related expression patterns found in this study, in which only *EoblCXE10* of the antennae-enriched *EoblCXE* genes displayed a male-specific expression pattern; the other *EoblCXEs* were either highly abundant in female antennae or were not sex-biased (Figure 3); however, even if then, this inference remains to be supported by the *in vitro* biochemical enzymatic experiment.

It should be noted that insect *CXE* genes belong to a multigene family that encodes sequence-divergent and functionally diverse proteins (Oakeshott et al., 1999; Tsubota and Shiotsuki, 2010). The 35 candidate EoblCXEs, which show average amino acid identities lower than 30%, fall into at least 10 different subclades; seven of these clades, which contain 33 EoblCXEs, possess clearly conserved functional characteristic features of the α/β-hydrolase structure, such as the Ser-Glu-His catalytic triad and the nucleophilic elbow surrounding the active-site serine residue (Gly-X-Ser-X-Gly) (Figure 1, Table 2). These features suggest that most EoblCXEs are catalytically active and participate in the degradation of diverse biologically important compounds. Other CXEs fall into the neuroligin, neuroreceptor, or gliotactin clades, including EoblCXE19 in the neuroreceptor clade and EoblCXE32 among the neuroligins, and lack the crucial residue Ser responsible for catalytic activity (Table 2); thus, these CXEs are considered to be catalytically inactive and are mainly involved in neurological and developmental functions related to sensory processing (Biswas et al., 2008; Durand et al., 2017).

Ten EoblCXEs belonging to the dipteran microsomal α-esterase or mitochondrial and cytosolic esterase clade lack a predicted signal peptide, indicating that they are intracellular esterases. These clades (particularly the α-esterases) are well-known for their involvement in the detoxification of insecticides and xenobiotics and the digestion of dietary esters (Newcomb et al., 1997; Campbell et al., 2003; Liang et al., 2007; Tang et al., 2011; Yang et al., 2016b; Gong et al., 2017). An orthologous gene of CXE10 has been functionally studied in two closely related *Spodoptera* species, *S. littoralis* (SlittCXE10) and *S. exigua* (SexiCXE10) (Durand et al., 2010a; He et al., 2015). Both SlittCXE10 and SexiCXE10 are reportedly expressed in the olfactory sensilla and preferentially degrade an ester plant volatile, (*Z*)-3-hexenyl acetate. EoblCXE10 shares high amino acid identity with SlittCXE10 and SexiCXE10 and is therefore considered an orthologous gene in *E. obliqua*. Its tissue expression was found to be restricted to the antennae of adults, particularly adult males, and resembled the distribution of its orthologs in *Spodoptera* species (Figure 3), implying a similar role in the degradation of host plant volatile compounds.

Approximately half of the candidate EoblCXEs clustered in the moth antennal esterase or ß-esterase and pheromone esterase clade and presented both a catalytically active Ser residue and a predicted signal peptide (Table 2). These CXEs represented typical secreted or extracellular esterases that could be secreted into the sensillum lymph surrounding the sensory neurons or into the hemolymph filling the antennal lumen, indicating potential roles in the degradation of odorants and the maintenance of OSN sensitivity. Indeed, functional reports regarding several members of these clades, such as ApolPDE (Vogt and Riddiford, 1981; Ishida and Leal, 2005), PjapPDE (Ishida and Leal, 2008), SexiCXE13 (He et al., 2014a), SexiCXE14 (He et al., 2014c), and SlittCXE7 (Durand et al., 2011), indicate that they play crucial roles in the degradation of insect sex pheromones and biologically important plant volatiles.

To gain further insight into the physiological roles of the EoblCXEs involved in hydrolyzing tea plant volatiles in *E. obliqua* olfaction, we selected *EoblCXE7* and *EoblCXE13* for a fluorescence *in situ* hybridization assay, not only because *EoblCXE7* and *EoblCXE13* are comparably expressed in the antennae of both *E. obliqua* sexes but also because EoblCXE13 clusters with ApolPDE, PjapPDE and SexiCXE13, while EoblCXE7 falls into the same clade as SlittCXE7 and SexiCXE14; therefore, these genes provide a useful model for comparative analyses of the functional evolution of CXE orthologs across lepidopteran species. In contrast to *SlittCXE7* (Durand et al., 2011), the localization of *EoblCXE7* and *EoblCXE13* at the sensillum level is more complex. *EoblCXE7* exhibits a similar cellular localization at Str and Sba between the sexes, whereas *EoblCXE13* is extensively expressed at multiple sensilla of female *E. obliqua* but is mainly restricted to Str I in males. Interestingly, (*Z*)-3-hexenyl acetate, a substrate of both CXE13 and CXE7 in *Spodoptera* species (Durand et al., 2011; He et al., 2014a), can attract both virgin males and mated females in *E. obliqua*. These results suggested that EolbCXE7 and EoblCXE13 might function in the *E. obliqua* olfactory system; however, whether the functions of EolbCXE7 and EoblCXE13 resemble those of their orthologs SlittCXE7 and SexiCXE13 as candidate ODEs in the degradation of (*Z*)-3-hexenyl acetate remains to be further confirmed. Additionally, *EoblCXE7* and *EoblCXE13* were highly expressed at Sst, a putative gustatory sensillum in lepidopteran species (Zenker et al., 2011; Tang et al., 2015), and we therefore cannot rule out the possibility that both of these *EoblCXEs* might function in the gustatory system.

In summary, the present study provides the first identification and characterization of the expression patterns of candidate CXE genes in *E. obliqua*, a common lepidopteran insect pest of the Geometridae, which will aid the development of new pest management techniques using CXE as potential targets for the disruption of insect foraging behavior.

## Author contributions

LS, QianW, and YoZ conceived and designed the experimental plan. LS, QianW, and QiW preformed the experiments. LS, QianW, YuZ, MT, HG, JF, QX, and YaZ analyzed the data. LS and QianW drafted the manuscript.

### Conflict of interest statement

The authors declare that the research was conducted in the absence of any commercial or financial relationships that could be construed as a potential conflict of interest.

## Supplementary material

Figure S1 Sense probe control for *in situ* hybridization with biotin-labeled probes.

Figure S2 Original image of Figure 2.

Table S1 Accession numbers and full names of the insect CXEs used in the phylogenetic analysis.

Table S2 Primers employed in the RT-PCR assay.

Table S3 Primers used in the qPCR assay.

Table S4 Amino acid identities of EoblCXEs.

Table S5 The *t* and *p* values of Student's *t*-test.
