# Supplementary material for: Identification and Expression Patterns of Putative Diversified Carboxylesterases in the Tea Geometrid Ectropis obliqua Prout
Source: Front Physiol. 2017 Dec 18;8:1085. doi: 10.3389/fphys.2017.01085 (PMC5741679; doi:10.3389/fphys.2017.01085)
Supplement: Figure S1 — Sense probe control for in situ hybridization with biotin-labeled probes. [file DataSheet1.zip › Supplementary material/Table S1.docx]

**Table S1.** Accession numbers and the full names of the insect CXEs used in the phylogenetic analysis.

| **CXE name** | **Species** | **Accession number** |
| --- | --- | --- |
| SinfCXE1 | *Sesamia inferens* | KF960776 |
| SinfCXE3 | *Sesamia inferens* | KF960778 |
| SinfCXE5 | *Sesamia inferens* | KF960779 |
| SinfCXE6 | *Sesamia inferens* | KF960780 |
| SinfCXE9 | *Sesamia inferens* | KF960781 |
| SinfCXE10 | *Sesamia inferens* | KF960782 |
| SinfCXE11 | *Sesamia inferens* | KF960783 |
| SinfCXE12 | *Sesamia inferens* | KF960784 |
| SinfCXE13 | *Sesamia inferens* | KF960785 |
| SinfCXE14 | *Sesamia inferens* | KF960786 |
| SinfCXE16 | *Sesamia inferens* | KF960787 |
| SinfCXE18 | *Sesamia inferens* | KF960788 |
| SinfCXE19 | *Sesamia inferens* | KF960789 |
| SinfCXE20 | *Sesamia inferens* | KF960790 |
| SinfCXE26 | *Sesamia inferens* | KF960791 |
| SlittCXE20 | *Spodoptera littoralis* | FJ652463 |
| SlittCXE19 | *Spodoptera littoralis* | FJ652462 |
| SlittCXE18 | *Spodoptera littoralis* | FJ652461 |
| SlittCXE17 | *Spodoptera littoralis* | FJ652460 |
| SlittCXE16 | *Spodoptera littoralis* | FJ652459 |
| SlittCXE15 | *Spodoptera littoralis* | FJ652458 |
| SlittCXE14 | *Spodoptera littoralis* | FJ652457 |
| SlittCXE13 | *Spodoptera littoralis* | FJ652456 |
| SlittCXE12 | *Spodoptera littoralis* | FJ652455 |
| SlittCXE11 | *Spodoptera littoralis* | FJ652454 |
| SlittCXE10 | *Spodoptera littoralis* | FJ652453 |
| SlittCXE9 | *Spodoptera littoralis* | FJ652452 |
| SlittCXE8 | *Spodoptera littoralis* | FJ652451 |
| SlittCXE7 | *Spodoptera littoralis* | FJ652450 |
| SlittCXE6 | *Spodoptera littoralis* | FJ652449 |
| SlittCXE5 | *Spodoptera littoralis* | FJ652448 |
| SlittCXE4 | *Spodoptera littoralis* | FJ652447 |
| SlittCXE3 | *Spodoptera littoralis* | FJ652446 |
| SlittCXE2 | *Spodoptera littoralis* | FJ652445 |
| SlittEst | *Spodoptera littoralis* | DQ680828 |
| CG2505aE2 | *Drosophila melanogaster* | NM_079544 |
| CG1031aE1 | *Drosophila melanogaster* | NM_079545 |
| CG6018aE1-0 | *Drosophila melanogaster* | NM_137834 |
| CG1089aE5 | *Drosophila melanogaster* | NM_001260036 |
| CG1112aE7 | *Drosophila melanogaster* | NM_079537 |
| CG1121aE8 | *Drosophila melanogaster* | NM_079535 |
| CG1128aE9 | *Drosophila melanogaster* | NM_169187 |
| CG1131aE10 | *Drosophila melanogaster* | NM_001260034 |
| CG1257aE3 | *Drosophila melanogaster* | NM_079543 |
| CG1108aE6 | *Drosophila melanogaster* | BT044260 |
| CG1082aE4 | *Drosophila melanogaster* | NM_079542 |
| CG9858 | *Drosophila melanogaster* | NM_080523 |
| CG10175 | *Drosophila melanogaster* | NM_142894 |
| TcasEst | *Tribolium castaneum* | XM_008197262 |
| BmorJHE | *Bombyx mori* | NM_001043562 |
| MsexJHE | *Manduca sexta* | AF327882 |
| HvirJHE | *Heliothis virescens* | J04955 |
| SnonEst | *Sesamia nonagrioides* | DQ680829 |
| ApolODE | *Antheraea polyphemus* | AY091503 |
| MbraEst | *Mamestra brassicae* | AY390258 |
| AploIE | *Antheraea polyphemus* | AY091504 |
| GB16889 | *Apis mellifera* | XM_393293 |
| CG4382 | *Drosophila melanogaster* | NM_135457 |
| CG4757 | *Drosophila melanogaster* | NM_141786 |
| CG3841 | *Drosophila melanogaster* | NM_135456 |
| GB10820 | *Apis mellifera* | NM_001126244 |
| GB15327 | *Apis mellifera* | NM_001011563 |
| PjapPDE | *Popillia japonica* | AY866482 |
| ApolPDE | *Antheraea polyphemus]* | AY866480 |
| CG6917 | *Drosophila melanogaster* | NM_176322 |
| CG17148 | *Drosophila melanogaster* | NM_176323 |
| MperMFE4 | *M. persicae* | X74555 |
| NlugCXE | *Nilaparvata lugens* | AF302777 |
| CG6414 | *Drosophila melanogaster* | BT150265 |
| GB11403 | *Apis mellifera* | XM_391943 |
| TmolJHE | *Tenebrio molitor* | AF448479 |
| CG8425JHE | *Drosophila melanogaster* | NM_079034 |
| CG8424 | *Drosophila melanogaster* | NM_137241 |
| GB18720 | *Apis mellifera* | XM_006561837 |
| GB10066 nlg1 | *Apis mellifera* | XM_006571491 |
| GB13939 | *Apis mellifera* | NM_001145739 |
| GB18836 | *Apis mellifera* | XM_006561556 |
| GB18290 | *Apis mellifera* | NM_001145736 |
| CG10339 | *Drosophila melanogaster* | NM_138037 |
| GB18901 | *Apis mellifera* | XM_016912947 |
| CG3903 | *Drosophila melanogaster* | NM_057254 |
| GB12309 | *Apis mellifera* | XM_396706 |
| NlugJHE | *Nilaparvata lugens* | EU380769 |
| PhilJHE | *Psacothea hilaris* | AB259898 |
| GassJHE | *Gryllus assimilis* | EF558769 |
| AaegJHE | *Aedes aegypti* | CH477330 |
| CfumJHE | *Choristoneura fumiferana* | AF153367 |
| SexiCXE5 | *Spodoptera exigua* | HQ116561 |
| SexiCXE10 | *Spodoptera exigua* | JF728805 |
| SexiCXE11 | *Spodoptera exigua* | JF728804 |
| SexiCXE13 | *Spodoptera exigua* | HQ116560 |
| SexiCXE14 | *Spodoptera exigua* | JF728803 |
| SexiCXE17 | *Spodoptera exigua* | HQ116559 |
| SexiCXE18 | *Spodoptera exigua* | JF728802 |
| SexiCXE20 | *Spodoptera exigua* | HQ116562 |
| SlituCXE13 | *Spodoptera litura* | HQ116556 |
| SlituCXE17 | *Spodoptera litura* | HQ116558 |
| SlituCXE18 | *Spodoptera litura* | HQ116557 |
| EoblCXE1 | *Ectropis obliqua* | KX015843 |
| EoblCXE2 | *Ectropis obliqua* | KX015844 |
| EoblCXE3 | *Ectropis obliqua* | KX015845 |
| EoblCXE4 | *Ectropis obliqua* | KX015846 |
| EoblCXE5 | *Ectropis obliqua* | KX015847 |
| EoblCXE6 | *Ectropis obliqua* | KX015848 |
| EoblCXE7 | *Ectropis obliqua* | KX015849 |
| EoblCXE8 | *Ectropis obliqua* | KX015850 |
| EoblCXE9 | *Ectropis obliqua* | KX015851 |
| EoblCXE10 | *Ectropis obliqua* | KX015852 |
| EoblCXE11 | *Ectropis obliqua* | KX015853 |
| EoblCXE12 | *Ectropis obliqua* | KX015854 |
| EoblCXE13 | *Ectropis obliqua* | KX015855 |
| EoblCXE14 | *Ectropis obliqua* | KX015856 |
| EoblCXE15 | *Ectropis obliqua* | KX015857 |
| EoblCXE16 | *Ectropis obliqua* | KX015858 |
| EoblCXE17 | *Ectropis obliqua* | KX015859 |
| EoblCXE18 | *Ectropis obliqua* | KX015860 |
| EoblCXE19 | *Ectropis obliqua* | KX015861 |
| EoblCXE20 | *Ectropis obliqua* | KX015862 |
| EoblCXE21 | *Ectropis obliqua* | KX015863 |
| EoblCXE22 | *Ectropis obliqua* | KX015864 |
| EoblCXE23 | *Ectropis obliqua* | KX015865 |
| EoblCXE24 | *Ectropis obliqua* | KX015866 |
| EoblCXE25 | *Ectropis obliqua* | KX015867 |
| EoblCXE26 | *Ectropis obliqua* | KX015868 |
| EoblCXE27 | *Ectropis obliqua* | KX015869 |
| EoblCXE28 | *Ectropis obliqua* | KX015870 |
| EoblCXE29 | *Ectropis obliqua* | KX015871 |
| EoblCXE30 | *Ectropis obliqua* | KX015872 |
| EoblCXE31 | *Ectropis obliqua* | KX015873 |
| EoblCXE32 | *Ectropis obliqua* | KX015874 |
| EoblCXE33 | *Ectropis obliqua* | KX015875 |
| EoblCXE34 | *Ectropis obliqua* | KX015876 |
| EoblCXE35 | *Ectropis obliqua* | KX015877 |
