# Supplementary material for: Identification and Expression Patterns of Putative Diversified Carboxylesterases in the Tea Geometrid Ectropis obliqua Prout
Source: Front Physiol. 2017 Dec 18;8:1085. doi: 10.3389/fphys.2017.01085 (PMC5741679; doi:10.3389/fphys.2017.01085)
Supplement: Figure S1 — Sense probe control for in situ hybridization with biotin-labeled probes. [file DataSheet1.zip › Supplementary material/Table S2.docx]

Table S2. The primers used in the RT-PCR assay

| *EoblCXE1*-RT-PCR-F | GGCATCCCCTACGGCACT |
| --- | --- |
| *EoblCXE1*-RT-PCR-R | CGTCGTCTGGGTTTCCTCC |
| *EoblCXE2*-RT-PCR-F | ATCCTCAGGGAAGGTGGC |
| *EoblCXE2*-RT-PCR-R | AGCCGTAAGGCCCTAGTCT |
| *EoblCXE3*-RT-PCR-F | AAGGCGGCGGCCAGTA |
| *EoblCXE3*-RT-PCR-R | CGGGAACGTCTTCCGTATCT |
| *EoblCXE4*-RT-PCR-F | GTGGTGGCTTGGCTTTCG |
| *EoblCXE4*-RT-PCR-R | CCATCGCAGTGCCGTTTT |
| *EoblCXE5*-RT-PCR-F | GAACGAAGAATGGCACCG |
| *EoblCXE5*-RT-PCR-R | CGTGTCTGGCGAAGTTGTAG |
| *EoblCXE6*-RT-PCR-F | TGGACGGCGTGAGGCA |
| *EoblCXE6*-RT-PCR-R | GGCGAGACTTTGAGGGGTTA |
| *EoblCXE7*-RT-PCR-F | GCCGAGGATGGGGTCT |
| *EoblCXE7*-RT-PCR-R | GTGCTGTGGCGTATGGG |
| *EoblCXE8*-RT-PCR-F | CTTGGGGCACTTGGTTTC |
| *EoblCXE8*-RT-PCR-R | GCTTTGGGCTATCGCTCT |
| *EoblCXE9*-RT-PCR-F | TGGAAGCAGCTCAGGAGG |
| *EoblCXE9*-RT-PCR-R | GGTGGGCAAGATCGGTAA |
| *EoblCXE10*-RT-PCR-F | ACCTGGACATCGGGAACC |
| *EoblCXE10*-RT-PCR-R | CACGGCATAGCTTGGGAG |
| *EoblCXE11*-RT-PCR-F | GGTTCGTCCCGATAGTTGAG |
| *EoblCXE11*-RT-PCR-R | CCGTGACATGCTCCCTTT |
| *EoblCXE12*-RT-PCR-F | GCAGAGGATGCCAAAGAGTC |
| *EoblCXE12*-RT-PCR-R | ATGTTGGAGGCGGAAGAG |
| *EoblCXE13*-RT-PCR-F | GCGACCAGCCAGGAGAT |
| *EoblCXE13*-RT-PCR-R | TAGGGGCAGGGTGTCATT |
| *EoblCXE14*-RT-PCR-F | GTATTAGGCGTTGTGGGTC |
| *EoblCXE14*-RT-PCR-R | CGGGGCAAAGAGCAGTG |
| *EoblCXE15*-RT-PCR-F | CTCGCCGCTGTATGCC |
| *EoblCXE15*-RT-PCR-R | CAAGTCGCTGCGTCAAGTCT |
| *EoblCXE16*-RT-PCR-F | TGTTTTGTGGGCGGCTCT |
| *EoblCXE16*-RT-PCR-R | GCGGTAGTTGGCGGTGA |
| *EoblCXE17*-RT-PCR-F | GCTCCAGGACCTGAACCG |
| *EoblCXE17*-RT-PCR-R | GCGAAGGCAAAGGAAACC |
| *EoblCXE18*-RT-PCR-F | GCCCCTGAGGTGGTAATCG |
| *EoblCXE18*-RT-PCR-R | CGCTCCGCCGTGAATGT |
| *EoblCXE19*-RT-PCR-F | CGGACGATTTCAAGGTGG |
| *EoblCXE19*-RT-PCR-R | TGACCCAGTAGAGGGATGCT |
| *EoblCXE20-*RT-PCR-F | CGGCTTCGGTTGTATTCC |
| *EoblCXE20*-RT-PCR-R | GCTATGGTCGTGTCGTTCTC |
| *EoblCXE21*-RT-PCR-F | CTCTTCCACCTCCGACAT |
| *EoblCXE21*-RT-PCR-R | GCACTACCGCCTTCAATG |
| *EoblCXE22*-RT-PCR-F | TTCAAGGCACCGCTACCC |
| *EoblCXE22*-RT-PCR-R | CACCCAACGCAACAAGG |
| *EoblCXE23*-RT-PCR-F | GGAACAGGAGCGTTTGGC |
| *EoblCXE23*-RT-PCR-R | CCTCGTCGGTGGTGCA |
| *EoblCXE24*-RT-PCR-F | CTTGCTGGGAGTGAGTGAGA |
| *EoblCXE24*-RT-PCR-R | GCGTGGGGTTGGGTTAC |
| *EoblCXE25*-RT-PCR-F | CGTATGCCGAGCCACC |
| *EoblCXE25*-RT-PCR-R | GCCCCAGCACTTTCACC |
| *EoblCXE26*-RT-PCR-F | TGCGAGGGCATTGGAA |
| *EoblCXE26*-RT-PCR-R | CGTGGTGGGGACAGGTTT |
| *EoblCXE27*-RT-PCR-F | AACGAAAGCCACTACGGTC |
| *EoblCXE27*-RT-PCR-R | CAATCGCACAACGGTACTCC |
| *EoblCXE28*-RT-PCR-F | CTTACTCATCGGTCACACTA |
| *EoblCXE28*-RT-PCR-R | TATTCAACTCCTGCGTCTT |
| *EoblCXE29*-RT-PCR-F | CCCCAACTTCACCACAACC |
| *EoblCXE29*-RT-PCR-R | CCCAGTCACAGAACGGCAC |
| *EoblCXE30*-RT-PCR-F | CTTACCAACCGACACAAC |
| *EoblCXE30*-RT-PCR-R | CATCTTCAGCCACTTAGC |
| *EoblCXE31*-RT-PCR-F | GGCAAAGGTGGAGGTCTCG |
| *EoblCXE31*-RT-PCR-R | TGGTCCGTAGTGCTGGTCGT |
| *EoblCXE32*-RT-PCR-F | GATTTGGTGCGTCGTGC |
| *EoblCXE32*-RT-PCR-R | AGGAGGCTCTGCGTATCTG |
| *EoblCXE33*-RT-PCR-F | GGAAAGGCAGCCGTCG |
| *EoblCXE33*-RT-PCR-R | CCCGCACATCCAACCAC |
| *EoblCXE34*-RT-PCR-F | AAGGTCAGCCAGCGTTCT |
| *EoblCXE34*-RT-PCR-R | CCATTCCCCTGCGTAGTTC |
| *EoblCXE35*-RT-PCR-F | GCTTGTGCCTGAATGTCCTG |
| *EoblCXE35*-RT-PCR-R | TCCCGGCTTCCTGTGC |
