# Supplementary material for: Identification and Expression Patterns of Putative Diversified Carboxylesterases in the Tea Geometrid Ectropis obliqua Prout
Source: Front Physiol. 2017 Dec 18;8:1085. doi: 10.3389/fphys.2017.01085 (PMC5741679; doi:10.3389/fphys.2017.01085)
Supplement: Figure S1 — Sense probe control for in situ hybridization with biotin-labeled probes. [file DataSheet1.zip › Supplementary material/Table S3.docx]

Table S3. The primers used in the qPCR assay

| EoblCXE2-qPCR-F | GCACACCTGACAACAAG |
| --- | --- |
| *EoblCXE2*-qPCR-R | TTCTATGACAATCTCCTCCAA |
| *EoblCXE3*-qPCR-F | AACTCGTCGCAACTGA |
| *EoblCXE3*-qPCR-R | CTCTTCGTCGGTGTGA |
| *EoblCXE5*-qPCR-F | ATAGATTGGCGAACCT |
| *EoblCXE5*-qPCR-R | CGAGAAGATGTAGATGAG |
| *EoblCXE6*-qPCR-F | CGAATCCACAAGAACAGA |
| *EoblCXE6*-qPCR-R | ATCACAACAACACTCAGAA |
| *EoblCXE7*-qPCR-F | GTTGAGGAAGTCGTTAGC |
| *EoblCXE7*-qPCR-R | CTGTGGCGTATGGGATA |
| *EoblCXE8*-qPCR-F | GACTATTGGAGGACGATT |
| *EoblCXE8*-qPCR-R | TTGCGGACATTGGTAAT |
| *EoblCXE10*-qPCR-F | GCTTCCTCTTACTCAATCT |
| *EoblCXE10*-qPCR-R | TCTCTTCAACACTTCAACA |
| *EoblCXE12*-qPCR-F | ATTATCGCAGTTACATTCAA |
| *EoblCXE12*-qPCR-R | TTTCATTCCAGCATTTCC |
| *EoblCXE13*-qPCR-F | GCGACCAGCCAGGAGAT |
| *EoblCXE13*-qPCR-R | CCGCAGCAGGGTAAAGG |
| *EoblCXE14*-qPCR-F | ACCAACGAAGATGTAACCAATG |
| *EoblCXE14*-qPCR-R | CAAGACGGCAGAGACGAA |
| *EoblCXE15*-qPCR-F | TGAGGGCTGCCACGAA |
| *EoblCXE15*-qPCR-R | CGCCGACCACTGAACTAAAA |
| *EoblCXE18*-qPCR-F | CCTATGCCACTACTAACA |
| *EoblCXE18*-qPCR-R | TTCGTCAACTGCTCTG |
| *EoblCXE20*-qPCR-F | AAAGTGCCTGTAATAATGGGATA |
| *EoblCXE20*-qPCR-R | CTATGGTCGTGTCGTTCTC |
| *EoblCXE22*-qPCR-F | TCTGTTGACCTTCTAATGC |
| *EoblCXE22*-qPCR-R | TAATGCGATGGCTCCTA |
| *EoblCXE23*-qPCR-F | GGTCATAGTCGTGGTTCA |
| *EoblCXE23*-qPCR-R | CATTCCTCAGCAAGTATTCG |
| *EoblCXE24*-qPCR-F | CCGCAACCAAACTCATCA |
| *EoblCXE24*-qPCR-R | CTCACTTCACTCGCTAACAG |
| *EoblCXE26*-qPCR-F | CTGTTCGGTCCTGTGATA |
| *EoblCXE26*-qPCR-R | GCTAAGTTGCCTCTGTTC |
| *EoblCXE35*-qPCR-F | CAGAGTCGTGAGAATGTG |
| *EoblCXE35*-qPCR-R | CGCCAATGTGAAGATAGT |
