# Supplementary material for: Identification and Expression Patterns of Putative Diversified Carboxylesterases in the Tea Geometrid Ectropis obliqua Prout
Source: Front Physiol. 2017 Dec 18;8:1085. doi: 10.3389/fphys.2017.01085 (PMC5741679; doi:10.3389/fphys.2017.01085)
Supplement: Figure S1 — Sense probe control for in situ hybridization with biotin-labeled probes. [file DataSheet1.zip › Supplementary material/Table S5.docx]

|  | ***t*** | ***p*** |
| --- | --- | --- |
| *EoblCXE2* | -0.4 | 0.729 |
| *EoblCXE5* | -8.77 | 0.0128 |
| *EoblCXE7* | -0.35 | 0.761 |
| *EoblCXE10* | 4.56 | 0.0449 |
| *EoblCXE12* | -0.34 | 0.7693 |
| *EoblCXE13* | -1.49 | 0.274 |
| *EoblCXE15* | -0.47 | 0.6828 |
| *EoblCXE22* | -0.14 | 0.8992 |
| *EoblCXE20* | -0.51 | 0.6579 |
| *EoblCXE24* | 0.37 | 0.7452 |

**Table S5.** The t and p values of Student’s t-test.
