# Supplementary figures and images for: Identification and Expression Patterns of Putative Diversified Carboxylesterases in the Tea Geometrid Ectropis obliqua Prout
Source: Front Physiol. 2017 Dec 18;8:1085. doi: 10.3389/fphys.2017.01085 (PMC5741679; doi:10.3389/fphys.2017.01085)

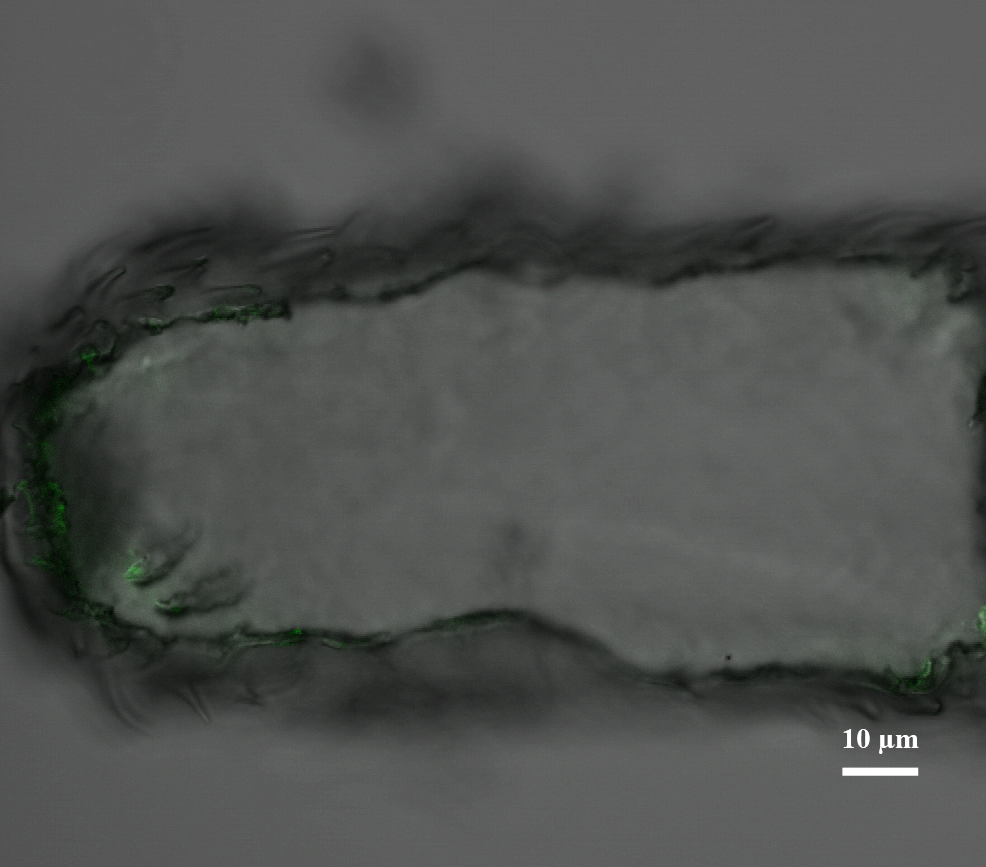

Supplement: Figure S1 — Sense probe control for in situ hybridization with biotin-labeled probes. [file DataSheet1.zip › Supplementary material/Figure S1.tif]

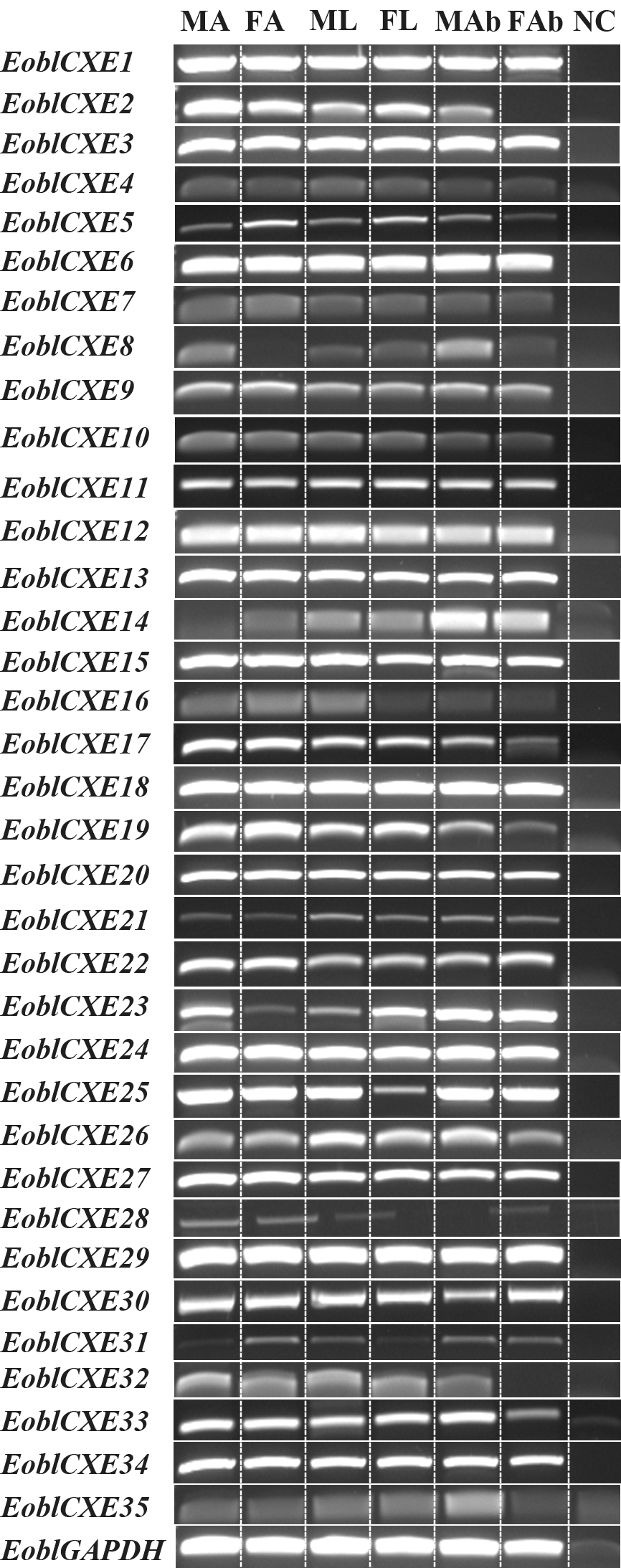

Supplement: Figure S2 — Original image of Figure 2. [file FigureS2.tif]
